# Pathological and oncological outcomes of pylorus-preserving versus conventional distal gastrectomy in early gastric cancer: a systematic review and meta-analysis

**DOI:** 10.1186/s12957-022-02766-0

**Published:** 2022-09-24

**Authors:** Sen Hou, Fan Liu, Zhidong Gao, Yingjiang Ye

**Affiliations:** 1 Department of Gastrointestinal Surgery, Peking University People’s Hospital, Beijing, 100044 People’s Republic of China; 2 Laboratory of Surgical Oncology, Peking University People’s Hospital, Beijing, 100044 People’s Republic of China; 3 Beijing Key Laboratory of Colorectal Cancer Diagnosis and Treatment Research, Peking University People’s Hospital, Beijing, 100044 People’s Republic of China

**Keywords:** Gastric cancer, Pylorus-preserving gastrectomy, Distal gastrectomy, Meta-analysis

## Abstract

**Background:**

Pylorus-preserving gastrectomy (PPG) is a function-preserving surgery for the treatment of early gastric cancer (EGC) in the middle third of the stomach. According to the literature reports, PPG decreases the incidence of dumping syndrome, bile reflux, gallstone formation, and nutritional deficit compared with conventional distal gastrectomy (CDG). However, the debates about PPG have been dominated by the incomplete lymphadenectomy and oncological safety. We carried out a systematic review and meta-analysis to evaluate the pathological and oncological outcomes of PPG.

**Methods:**

The protocol was registered in PROSPERO under number CRD42022304677. Databases including PubMed, Embase, Web of Science, and the Cochrane Register of Controlled Trials were searched before February 21, 2022. The outcomes included the pooled odds ratios (ORs) for dichotomous variables and weighted mean differences (WMDs) for continuous variables. For all outcomes, 95% confidence intervals (CIs) were calculated. Meta-analysis was performed using STATA software (Stata 14, Stata Corporation, Texas) and Review Manager 5.4.

**Results:**

A total of 4500 patients from 16 studies were included. Compared with the CDG group, the PPG group had fewer lymph nodes harvested (WMD= −3.09; 95% CI −4.75 to −1.43; *P* < 0.001). Differences in the number of resected lymph nodes were observed at stations No. 5, No. 6, No. 9, and No. 11p. There were no differences in lymph node metastasis at each station. Shorter proximal resection margins (WMD = −0.554; 95% CI −0.999 to −0.108; *P* = 0.015) and distal resection margins (WMD = −1.569; 95% CI −3.132 to −0.007; *P* = 0.049) were observed in the PPG group. There were no significant differences in pathological T1a stage (OR = 0.99; 95% CI 0.80 to 1.23; *P* = 0.88), T1b stage (OR = 1.01; 95% CI 0.81 to 1.26; *P* = 0.88), N0 stage (OR = 0.97; 95% CI 0.63 to 1.48; *P* = 0.88), tumor size (WMD = −0.10; 95% CI −0.25 to 0.05; *P* = 0.187), differentiated carcinoma (OR = 1.04; 95% CI 0.74 to 1.47; *P* = 0.812) or signet ring cell carcinoma (OR = 1.22; 95% CI 0.90 to 1.64; *P* = 0.198). No significant differences were observed between the groups in terms of overall survival (HR = 0.63; 95% CI 0.24 to 1.67; *P* = 0.852) or recurrence-free survival (HR = 0.29; 95% CI 0.03 to 2.67; *P* = 0.900).

**Conclusions:**

The meta-analysis of existing evidence demonstrated that the survival outcomes of PPG may be comparable to those of CDG. However, fewer lymph nodes at stations in No. 5, No. 6, No. 9, and No. 11p were harvested with PPG. We also found shorter proximal resection margins and distal resection margins for PPG, meaning more remnant stomachs would be preserved in PPG.

**Supplementary Information:**

The online version contains supplementary material available at 10.1186/s12957-022-02766-0.

Gastric cancer is the fifth most common cancer and the fourth leading cause of cancer mortality worldwide [1]. Early gastric cancer (EGC) is defined as gastric carcinoma confined to the mucosa and submucosa of the stomach, with or without regional lymph node metastasis (LNM) [2]. Due to the popularization of health screening programs, the proportion of EGC cases has been increasing [3, 4]. The treatment decision for EGC is complicated, diversified, and controversial. Endoscopic resection is established as first-line management for most EGC patients. However, gastrectomy with lymph node (LN) dissection remains the cornerstone of EGC management when risk factors are present, including LNM, lymphovascular invasion, submucosal invasion, poor differentiation, ulceration, and large tumor size [5–7].

Conventional distal gastrectomy (CDG) with lymphadenectomy substantially changes the anatomy of the normal stomach and causes functional and nutritional problems collectively known as “postgastrectomy syndromes” [8].

Pylorus-preserving gastrectomy (PPG) is a function-preserving gastrectomy for EGC located in the middle portion of the stomach, with a distance between the distal tumor border and the pylorus of 4 cm or greater [9]. The PPG technique reduces the extent of gastrectomy and retains the pyloric ring and the hepatic and pyloric branches of the vagal nerve [10, 11]. Therefore, PPG decreases the incidence of postgastrectomy syndromes, including dumping syndrome, bile reflux gastroesophagitis, gallstone formation, and nutritional deficits, compared with CDG [12–16].

Although PPG has superiority over CDG in terms of functional outcomes, PPG for EGC in the middle third of the stomach is weakly recommended [9]. In recent decades, the debates about PPG have been dominated by incomplete lymphadenectomy and oncological safety [17, 18]. The LNs, especially No. 5 and No. 6, could be incompletely dissected to save the nerve and artery [19–21]. A previous study suggested that PPG may be safe because the incidence of LNM in No. 5 and No. 6 was very low, 0–0.9% for No. 5 and 0–1.8% for No. 6 [19]. However, Wu et al. found that No. 3, 4, 5, and 6 LNs had the highest rates of metastasis for middle-third tumors. The incidence of LNM in No. 5 was as high as 3.05% [22]. The limited dissection of some regional LNs could increase the likelihood of recurrence [17, 19]. In addition, EGCs limited to the mucosa have a 2 to 5% incidence of LNM which increases to 10 to 25% when the disease invades the submucosa [23, 24]. For these reasons, the tumor invasion depth (pathological T stage) and proportion of positive LNs (pathological N stage) must be reappraised after surgery.

Our primary objective was to elucidate the number of LNs harvested and the precise distribution of LNM to each LN station. In addition, we aimed to elucidate the oncological safety of PPG. Our second objective was to evaluate the resection margin and pathological stages, tumor size, and histology of PPG in comparison with those of CDG.

## Materials and methods

### Protocol and registration

This systematic literature review and meta-analysis were reported in agreement with the Preferred Reporting Items for Systematic Reviews and Meta-analyses (PRISMA) guidelines [25]. No ethical approval or patient consent was required because all analyses were based on previously published studies. The protocol was registered in PROSPERO under number CRD42022304677 (available from https://www.crd.york.ac.uk/prospero/display_record.php?ID=CRD42022304677) (Additional file 1).

### Data sources and searches

A literature search of PubMed, Embase, and the Cochrane Register of clinical trials was carried out up until February 21, 2022, without language restrictions. Medical Subject Headings (MeSH) and free-text words were used, and the search items were as follows: pylorus preserving gastrectomy, gastric cancer, etc. Additionally, the reference lists of all of the articles included in the final analysis, as well as previous reviews, were searched to ensure the identification of all relevant studies. Details of the literature search are shown in Additional file 2.

### Selection and exclusion criteria

We evaluated the identified studies against the following predetermined inclusion criteria:

1. Population: patients with a pathological diagnosis of primary EGC who were treated by gastrectomy with lymph node dissection.
2. Intervention: PPG was performed.
3. Comparator: CDG was performed.
4. Outcomes: pathological outcomes, including the total number of LNs harvested, the number of LNs harvested at each station, the incidence of LNM at each station, length of the distal resection margin (DRM), the length of the proximal resection margin (PRM), pathological T stage (pT), pathological N stage (pN), tumor size, and histology; oncological outcomes: overall survival (OS) and recurrence-free survival (RFS).

The exclusion criteria were as follows:

1. The cancer of the enrolled patients was not at an early stage or the patient had other malignant tumors.
2. Single-arm study of PPG.
3. Patients in the control group were treated with endoscopic resection, local resection, or total gastrectomy.
4. Review articles, case reports, letters to the editor, meeting abstracts, and comments.
5. Original studies lacking available data.

### Study selection and data extraction

Endnote X9 software was used to remove duplicates. After two reviewers independently screened the titles and abstracts of the initially identified literature, eligible trials were identified. A third reviewer was consulted to resolve any disagreements between the two screening authors.

The following data were extracted from each included study: study characteristics: (1) titles, (2) duration of the study, (3) countries, (4) study design, (5) sample size, (6) interventions, etc.; patient characteristics, including age and sex; pathological outcomes: (1) harvested lymph node and LNM at each station, (2) DRM (cm), (3) PRM (cm), (4) pathological T stage, (5) pathological N stage, (6) tumor size, (7) differentiated tumor, and (8) signet ring cell carcinoma; oncological outcomes: (1) OS and (2) RFS.

### Quality assessment

The methodological qualities of retrospective cohort studies (RCSs) were evaluated using the Newcastle–Ottawa Quality Assessment Scale (NOS) for the adequacy of selection, the comparability of the groups, and the adequacy of outcome assessment [26]. The quality of the randomized controlled trials (RCTs) was assessed using the Cochrane Collaboration’s risk of bias assessment tool (Review Manager 5.4) [27] (details in Additional file 3).

### Statistical analysis

The statistical analysis was performed, and forest plots were generated via STATA software (Stata 14, Stata Corporation). The pooled odds ratios (ORs) were calculated along with 95% confidence intervals (CIs) for dichotomous outcomes, and weighted mean differences (WMDs) were calculated for continuous outcomes. If studies provided the median for continuous variables, the mean and standard deviation (SD) were not given. We transformed the data to means and SDs according to the method proposed by Hozo et al. [28]. For survival outcomes, HR values and CIs were extracted directly if they were provided in the literature. If not, the data of the survival curve was extracted through the Engauge digitizer. Then, the pooled HR was calculated according to the Excel program file provided by Tierney et al. [29].

Statistical heterogeneity among studies was assessed by the *I*^2^ statistic. If *I*^2^ < 50%, we used a fixed-effects model, while if *I*^2^ > 50%, we chose the random-effects model [30]. An approximation of the guidelines for the interpretations of *I*^2^ from the Cochrane Collaboration Handbook regards 0–40% as negligible heterogeneity, 30–60% as moderate heterogeneity, 50–90% as substantial heterogeneity, and 75–100% as considerable heterogeneity [31]. Subgroup analyses were conducted to investigate the influence on the overall results and discover the source of heterogeneity. Moreover, funnel plots and Egger’s test were generated to assess the publication bias of the included studies [32].

## Results

**Fig. 1:**
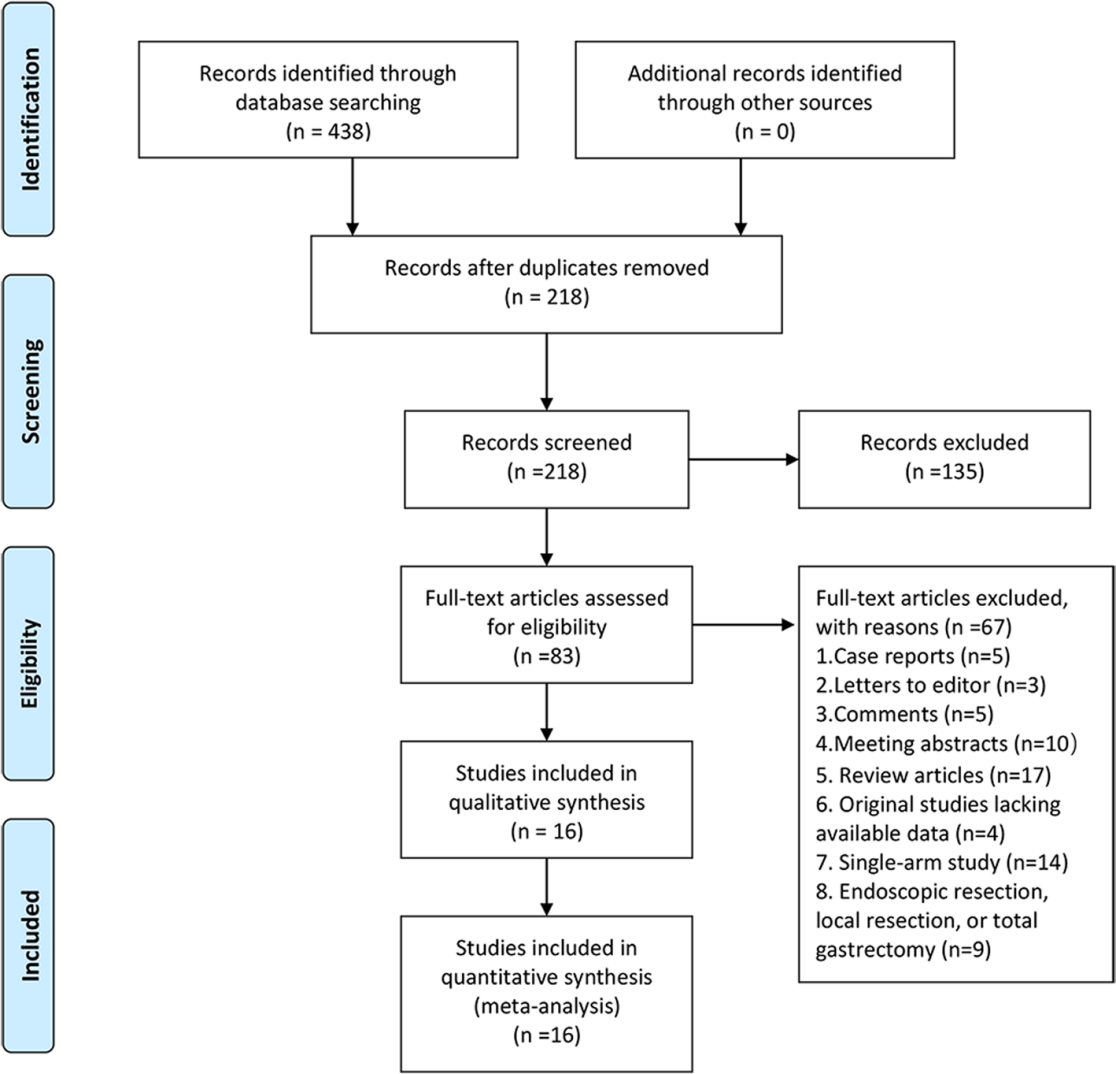
PRISMA flow chart of studies selection

Our initial search strategies yielded 438 studies, of which 220 were excluded after the abstract and method were screened. After a detailed, full-text read of 218 articles, 16 studies [14, 17, 19, 33–45] and 4500 patients were ultimately eligible for inclusion in the meta-analysis. Figure 1 shows a PRISMA diagram of the search flow in detail. The key characteristics of the included studies are listed in Table 1.

**Table 1:** Demographic of the included studies

Study, year	Country	Duration	Study design	Group	Method	Patients	Age (year)	Sex(male/female)	Pyloric cuff (cm)	Nerve preserved in PPG	Reconstructions	Lymphadenectomy D0/D1/D1+ /D2/D3
Zhang, 1998 [33]	China	1993–1995	RCS	PPG	NR	15	58.9 (9.4)	11/4	1.5	PB	Gastrogastrostomy	NR
				CDG	NR	28	58.0 (17.1)	21/7			B-I	NR
Shibata, 2004 [34]	Japan	1994–1996	RCT	PPG	NR	36	64 (1)	23/13	1.5	PB	Gastrogastrostomy	2/4/25/5/0
				CDG	NR	38	60 (2)	25/13			NR	1/13/19/5/0
Kong, 2009 [19]	Korea	2003–2008	RCS	PPG	NR	64	NR	NR	NR	NR	NR	NR
				CDG	NR	1380	NR	NR			NR	NR
Ikeguchi, 2010 [35]	Japan	1997–2007	RCS	PPG	O/Lap	46	62.8 (NR)	24/22	NR	HB PB	Gastrogastrostomy	NR
				CDG	O/Lap	87	64.2 (NR)	56/31			B-I	NR
Kim, 2014 [17]	Korea	2006–2012	RCS	PPG	NR	21	51.95 (NR)	13/8	NR	NR	NR	NR
				CDG	NR	109	55.8 (NR)	68/41			NR	NR
Suh, 2014 [36]	Korea	2003–2011	RCS	PPG	Lap	116	54.1 (12.3)	55/61	NR	HB	Gastrogastrostomy	NR
				CDG	Lap	176	59.1 (12.0)	107/69			B-I B-II RY	NR
Hu, 2015 [37]	China	2004–2009	RCS	PPG	NR	46	56.3 (11.4)	28/18	NR	HB	Gastrogastrostomy	NR
				CDG	NR	85	60.1 (12.3)	55/30			B-I	NR
Hu, 2016 [38]	China	2003–2010	RCS	PPG	Lap	35	55.0 (10.5)	19/16	NR	HB CB	Gastrogastrostomy	0/4/21/8/2
				CDG	Lap	25	60.3 (13.0)	15/10			B-I	0/8/10/6/1
Aizawa, 2017 [39]	Japan	2006–2012	RCS	PPG	NR	502	60.7 (9.6)	301/201	NR	CB	Gastrogastrostomy	0/502/0/0/0
				CDG	NR	502	61.7 (11.4)	309/193			B-I B-II RY	0/264/0/128/0
Hosoda, 2017 [14]	Japan	2006–2011	RCS	PPG	Lap	32	64.0 (9.5)	13/19	4.0	CB	Gastrogastrostomy	NR
				CDG	Lap	32	63.2 (8.8)	13/19			B-I	NR
Xia, 2018 [40]	China	2016–2017	RCS	PPG	Lap	46	57.9 (11.0)	23/23	3.0–4.0	HB	NR	NR
				CDG	Lap	66	53.0 (13.4)	35/31			NR	NR
Eom, 2019 [41]	Korea	2012–2015	RCS	PPG	Lap	101	58.3 (12.0)	54/47	3.0–5.0	HB PB	Gastrogastrostomy	NR
				CDG	Lap	195	56.5 (11.8)	114/81			B-II	NR
Xia, 2019 [42]	China	2015–2017	RCS	PPG	Lap	70	56.8 (10.9)	46/24	>3.0	HB CB	Gastrogastrostomy	NR
				CDG	Lap	97	57.5 (12.1)	63/34			B-I	NR
Zhu, 2019 [43]	Korea	2013–2016	RCS	PPG	NR	145	NR	67/78	3.0–5.0	HB	NR	NR
				CDG	NR	61	NR	34/27			NR	NR
Huang, 2020 [44]	China	2015–2017	RCS	PPG	Lap	40	60.5 (11.0)	25/15	>3.0	HB	Gastrogastrostomy	NR
				CDG	Lap	51	62.8 (10.2)	39/12			B-I	NR
Park, 2021 [45]	Korea	2015–2017	RCT	PPG	Lap	124	55.6 (10.6)	58/66	4.1 (0.9)	HB CB	Gastrogastrostomy	0/0/124/0/0
				CDG	Lap	129	58.1 (10.2)	67/62			B-I B-II RY	0/0/127/2/0

Continuous variables are recorded as mean (SD)

*RCS* retrospective cohort study, *RCT* randomized controlled trial, *PPG* pylorus preserving gastrectomy, *CB* celiac branch, *HB* hepatic branch, *PB* pyloric branch, *O* open surgery, *Lap* laparoscopic surgery, *NR* not reported, *B-I* Billroth-I reconstruction, *B-II* Billroth-II reconstruction, *RY* Roux-en-Y reconstruction, *D1*, No. 1, 3, 4sb, 4d, 5, 6, 7; *D1+*, D1 + No. 8a, 9; *D2*, D1 + No. 8a, 9, 11p, 12a

### Lymph nodes harvested and metastasis

**Fig. 2:**
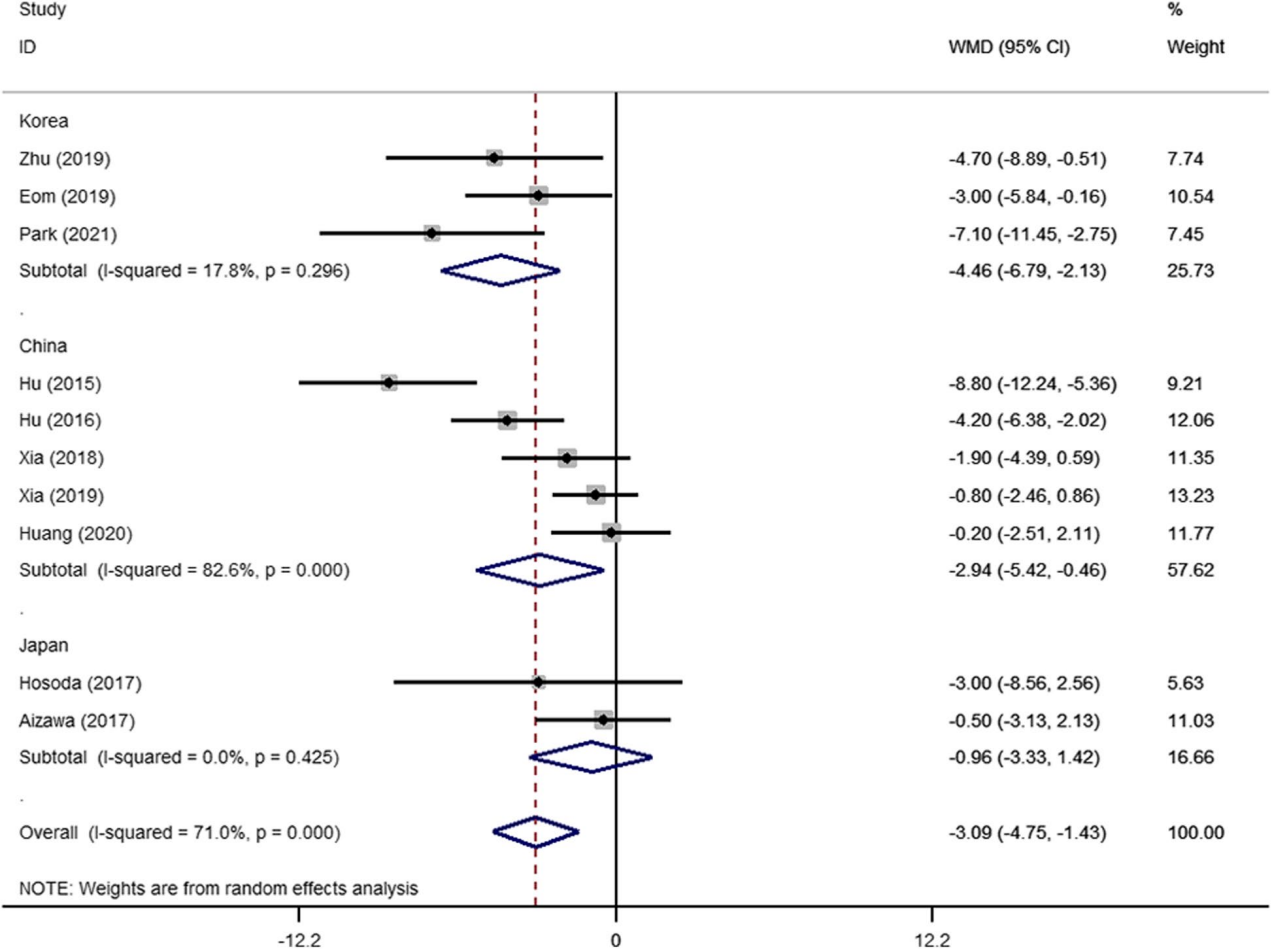
Subgroup analysis of LNs harvested according to countries

**Fig. 3:**
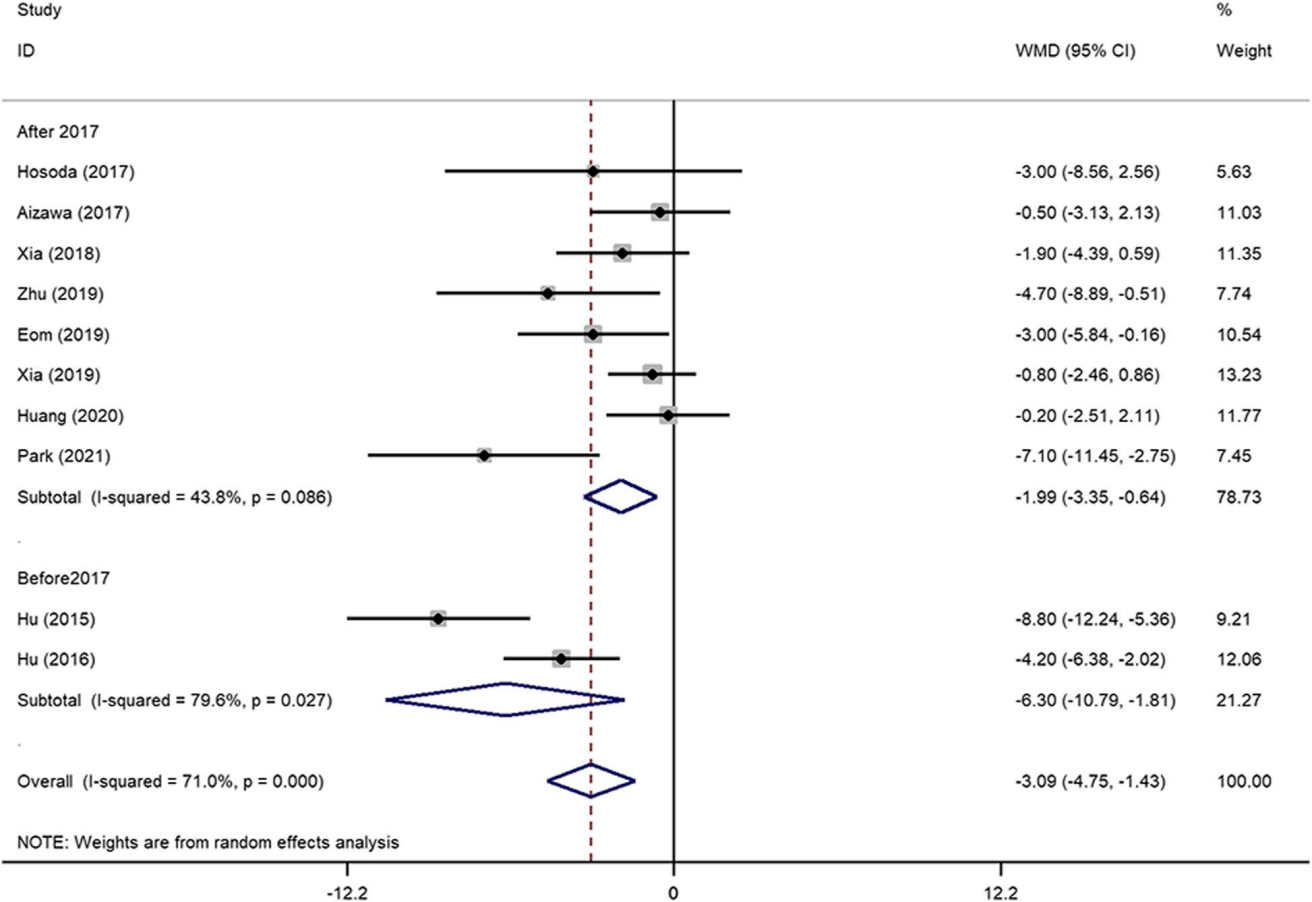
Subgroup analysis of LNs harvested according to publication years

The number of LNs harvested was reported in 10 studies [14, 37–45]. The pooled result was significantly different between the two operative approaches (WMD = −3.09; 95% CI −4.75 to −1.43; *P* < 0.001). However, the heterogeneity was substantial (*I*^2^ = 71.0%, *P*_heterogeneity_ < 0.001). We performed subgroup analysis according to three countries: China, Korea, and Japan. The pooled results revealed that obvious heterogeneity in Chinese studies (*I*^2^ = 82.6%, *P*_heterogeneity_ =0.001) but not in Japanese studies (*I*^2^ = 0%, *P*_heterogeneity_ = 0.425) or Korean studies (*I*^2^ = 17.8%, *P*_heterogeneity_ = 0.296) (Fig. 2). Similarly, we performed subgroup analysis according to the publication years. The heterogeneity was mainly from literatures before 2017 (*I*^2^ = 79.6%, *P*_heterogeneity_ = 0.027) (Fig. 3).

The number of LNs harvested at station No. 5 was significantly lower for PPG than for CDG (WMD = −0.60; 95% CI −0.77 to −0.43; *P* < 0.001). The quality of the pooled result was low because of the significantly unexplained heterogeneity among the included studies (*I*^2^ = 94%, *P*_heterogeneity_ < 0.001). The number of No. 6 LNs harvested was reported in five studies [14, 17, 19, 43, 45] and was lower for PPG than CDG (WMD = −0.52; 95% CI −0.99 to −0.04; *P* = 0.03). The number of LNs harvested at stations No. 9 and No.11p in the PPG group was also less than the number harvested in the CDG group, with low heterogeneity (Table 2).

**Table 2:** Pooled results of LNs harvested at each station

Outcomes	No. of studies	No. of participants	Statistical method	Effect size	*P*	Heterogeneity	
						*I*^2^	*P*
Station 1	2 [43, 45]	459	Mean difference (IV, fixed, 95% CI)	−0.25 [−0.95, 0.44]	0.47	0%	0.40
Station 3	3 [19, 43, 45]	1903	Mean difference (IV, random, 95% CI)	−0.33 [−1.03, 0.36]	0.40	55%	0.11
Station 4sb	2 [43, 45]	459	Mean difference (IV, fixed, 95% CI)	0.23 [−0.23, 0.70]	0.32	0%	0.69
Station 4d	4 [14, 19, 43, 45]	1967	Mean difference (IV, fixed, 95% CI)	−0.41 [−1.07, 0.24]	0.22	0%	0.73
Station 5	4 [17, 19, 43, 45]	2000	Mean difference (IV, random, 95% CI)	−0.60 [−0.77, −0.43]	**<0.01**	94%	<0.001
Station 6	5 [14, 17, 19, 43, 45]	3127	Mean difference (IV, random, 95% CI)	−0.52 [−0.99, −0.04]	**0.03**	0%	0.90
Station 7	2 [43, 45]	459	Mean difference (IV, fixed, 95% CI)	−0.57 [−1.30, 0.15]	0.12	0%	0.34
Station 8a	2 [43, 45]	459	Mean difference (IV, fixed, 95% CI)	−0.07 [−0.64, 0.49]	0.80	0%	0.61
Station 9	2 [43, 45]	459	Mean difference (IV, fixed, 95% CI)	−0.66 [−1.15, −0.16]	**<0.01**	0%	0.84
Station 11p	2 [43, 45]	459	Mean difference (IV, fixed, 95% CI)	−0.76 [−1.21, −0.31]	**<0.01**	0%	0.83

The pooled results of LNM at each station were also compared between two different surgical procedures and no significant difference was observed (Table 3).

**Table 3:** Pooled results of LNM at each station

Outcomes	No. of studies	No. of participants	Statistical method	Effect size	*P*	Heterogeneity	
						*I*^2^	*P*
Station 1	2 [36, 43]	491	Odds ratio (M-H, fixed, 95% CI)	1.32 [0.33, 5.35]	0.70	0%	0.40
Station 3	2 [36, 43]	492	Odds ratio (M-H, fixed, 95% CI)	0.98 [0.38, 2.51]	0.96	0%	1.00
Station 4sb	2 [36, 43]	491	Odds ratio (M-H, fixed, 95% CI)	0.42 [0.03, 6.77]	0.54	NA	NA
Station 4d	2 [36, 43]	491	Odds ratio (M-H, fixed, 95% CI)	1.13 [0.38, 3.37]	0.82	0%	0.67
Station 5	3 [17, 36, 43]	384	Odds ratio (M-H, fixed, 95% CI)	1.68 [0.07, 42.69]	0.75	NA	NA
Station 6	3 [17, 36, 43]	621	Odds ratio (M-H, fixed, 95% CI)	1.42 [0.40, 5.05]	0.59	70%	0.04
Station 7	2 [36, 43]	490	Odds ratio (M-H, fixed, 95% CI)	1.64 [0.57, 4.76]	0.36	45%	0.18
Station 8	2 [36, 43]	471	Odds ratio (M-H, fixed, 95% CI)	0.83 [0.12, 5.95]	0.85	0%	0.52
Station 9	2 [36, 43]	469	Odds ratio (M-H, fixed, 95% CI)	0.84 [0.15, 4.72]	0.84	NA	NA
Station 11p	2 [36, 43]	426	Odds ratio (M-H, fixed, 95% CI)	2.39 [0.25, 23.04]	0.45	0%	0.59

### Proximal resection margin (PRM) and distal resection margin (DRM)

Six studies [36, 40, 42–45] reported the PRM using a randomized model. Compared with PPG, CDG achieved a greater PRM (WMD = −0.55; 95% CI −1.00 to −0.11; *P* = 0.015), but with high heterogeneity among the included studies (*I*^2^ = 85.1%, *P*_heterogeneity_ < 0.001).

**Fig. 4:**
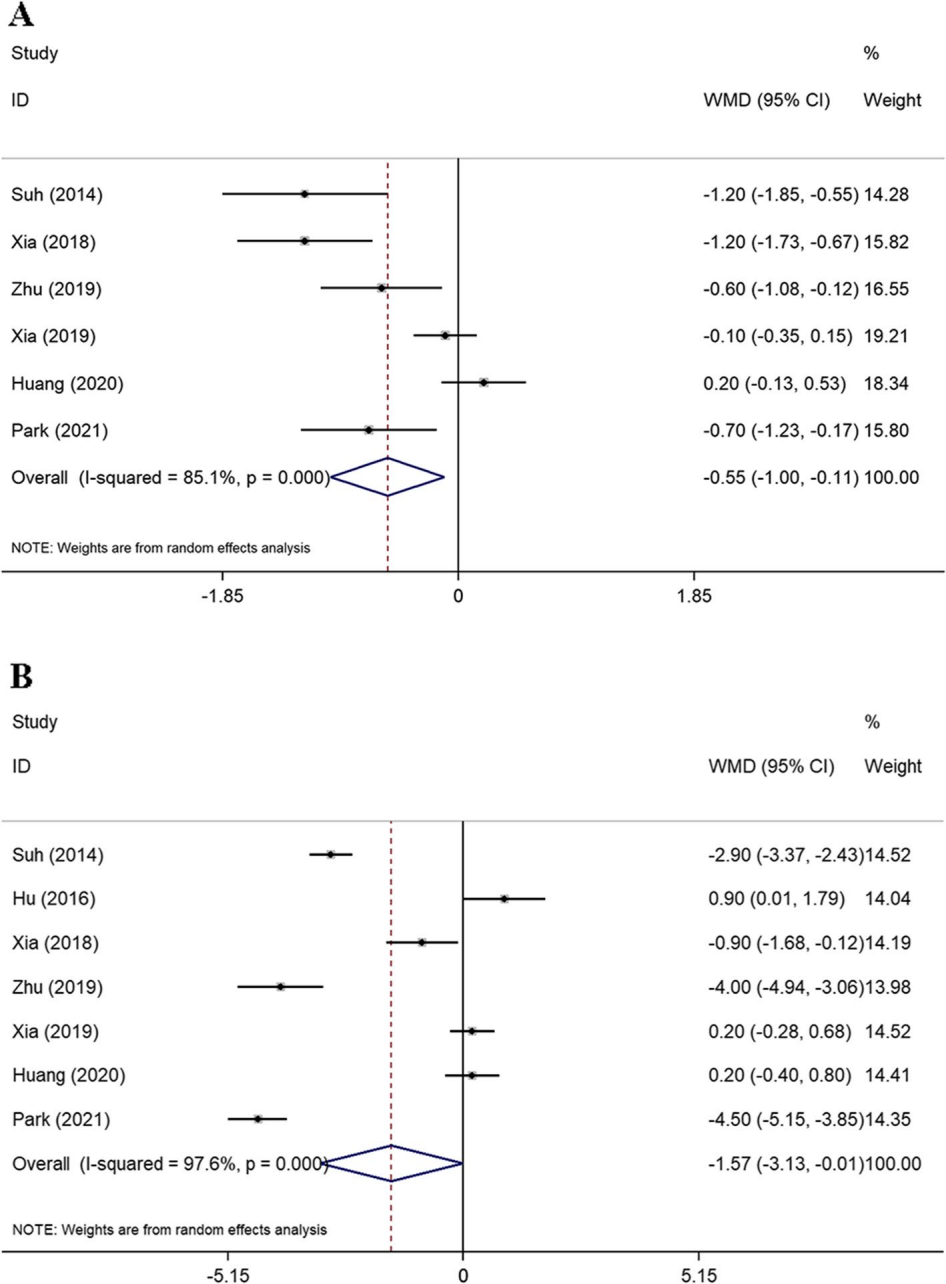
Forest plots for the meta-analysis of surgical margins. **A** PRM. **B** DRM

Seven studies [36, 38, 40, 42–45] reported the DRM. According to the analysis, CDG had a greater DRM than PPG (WMD = −1.57; 95% CI −3.13 to −0.01; *P* = 0.049). However, the heterogeneity was high (*I*^2^ = 97.6%, *P*_heterogeneity_ < 0.001) (Fig. 4).

### T stage and N stage

**Fig. 5:**
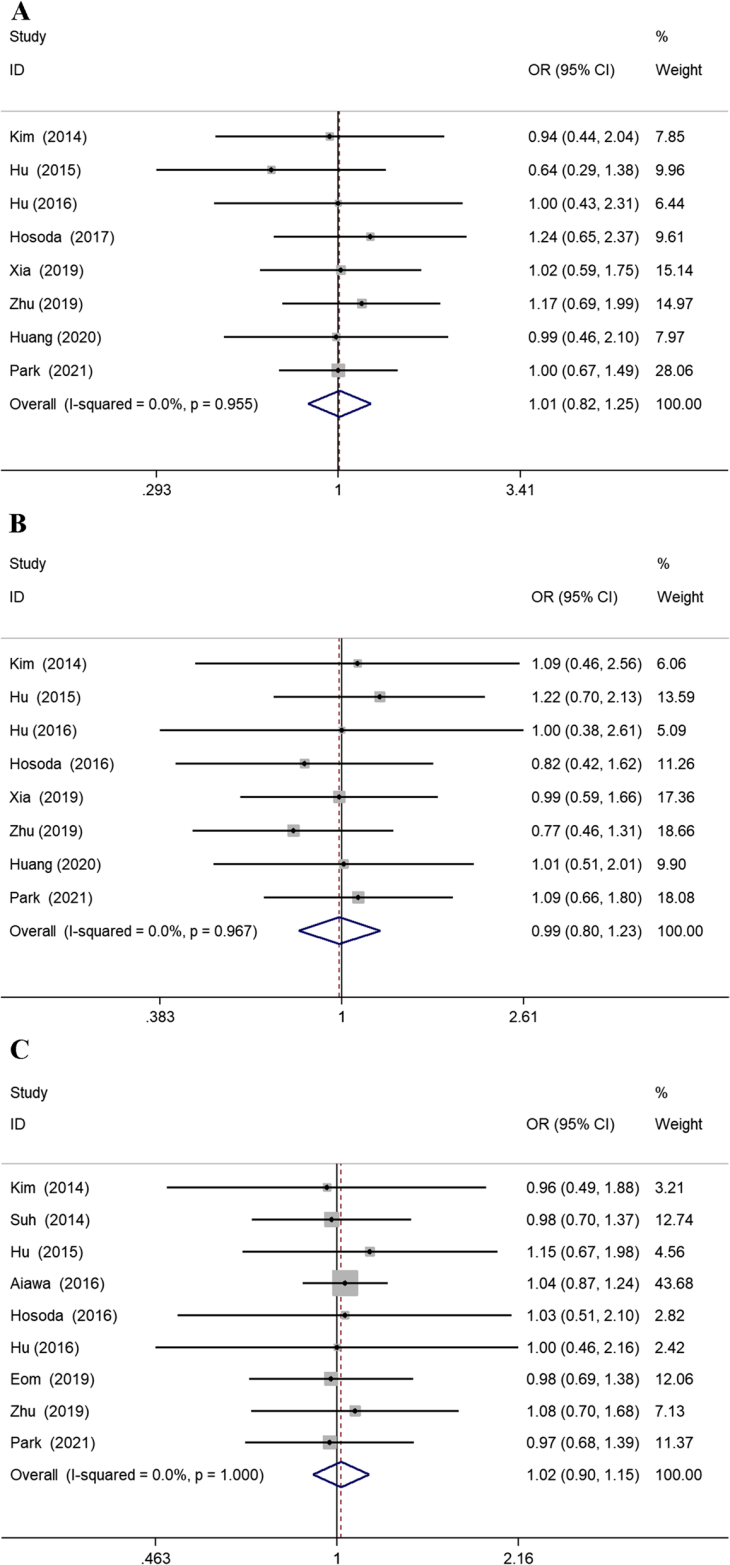
Forest plots for the meta-analysis of pathological T stages and N stages. **A** pT1a. **B** pT1b. **C** pN0

Eight studies [14, 17, 37, 38, 42–45] reported detailed pathological T stage, and the pooled results revealed no difference in pT1a and pT1b. Nine studies [14, 17, 36–39, 41, 43, 45] reported pathological N stage. There was no difference in the proportion of pN0 stage between PPG and CDG (OR =0.97; 95% CI 0.63 to 1.48; *P* = 0.88) (Fig. 5).

### Tumor size and histology

No significant differences were observed between the groups in terms of tumor size (WMD = −0.10; 95% CI −0.25 to 0.05; *P* = 0.187), differentiated carcinoma (OR = 1.04; 95% CI 0.74 to 1.47; *P* = 0.812) or signet ring cell carcinoma (OR = 1.22; 95% CI 0.90 to 1.64; *P* = 0.198) (Additional file 4).

### OS and RFS

**Fig. 6:**
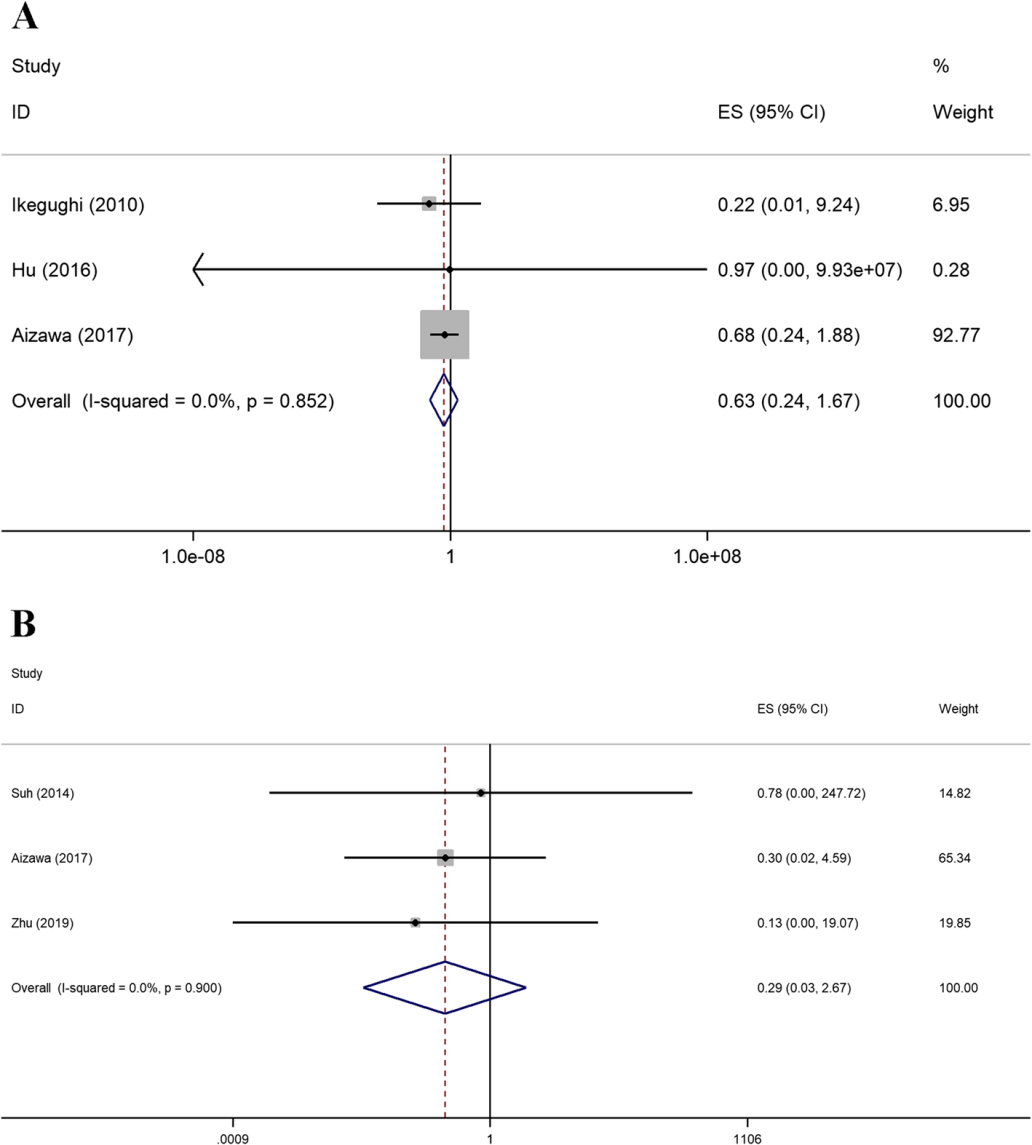
Forest plot for the meta-analysis of oncological outcomes. **A** OS. **B** RFS

For survival outcomes, none of the included literature reported the HRs or CIs of OS or RFS directly. Three publications provided survival curves of OS [35, 38, 39], and three studies reported survival curves of RFS [36, 39, 43]. The pooled results showed that there was no significant difference in OS (HR = 0.63; 95% CI 0.24 to 1.67; *P* = 0.852) or RFS (HR = 0.29; 95% CI 0.03 to 2.67; *P* = 0.900) between the two groups (Fig. 6).

### Publication bias

Funnel plots were generated to assess the publication bias of the included studies, and the results showed that no publication bias was found (Additional file 5).

## Discussion

In PPG, D1 lymphadenectomy includes stations No. 1, No. 3, No. 4sb, No. 4d, No. 6, and No. 7. Stations No. 8a and No. 9 are additionally included for D1+ lymphadenectomy. D2 lymphadenectomy was defined as D1+ resection combined with No. 11p and No. 12a resections [9]. Our meta-analysis showed that compared to CDG, PPG harvested fewer lymph nodes. Differences in the number of resected LNs were observed at stations No. 5, No. 6, No. 9, and No. 11p. The possible reasons were as follows. At station No. 5, the root of the right gastric artery and vein and the supra-pyloric lymph nodes were routinely left intact for PPG [46]. For PPG, station No. 6 was included in the lymphadenectomy, but for the purpose of protecting the infra-pyloric artery (IPA) in PPG; the range of dissection at No. 6 may be not as sufficient as that in CDG. The IPA diverges independently with the right gastroepiploic artery in 76.9 % of patients and is closely associated with a certain number of LNs, namely No. 6i [47]. The right gastroepiploic vessels were transected after the bifurcation of the infra-pyloric vessels, so lymph node dissection at No. 6i was achieved with some limitations [48]. The insufficient number of lymph nodes harvested at No. 9 and No. 11p with PPG may be due to the attempt to protect the vagus nerve with this method, which affects the surgical field or scope of the dissection. The heterogeneity among studies in the total LNs harvested was significant in our pooled results. Subgroup analysis showed that heterogeneity was mainly from the literature before 2017. The possible reason is that laparoscopic PPG remains technically demanding, and preserving the IPA in laparoscopic surgery requires a high degree of surgical skill. Subgroup analysis also showed differences in the number of LNs dissected for Chinese studies, probably because the surgical techniques and pathological detection abilities have not developed concurrently in China.

LNM is a definite poor prognostic factor for EGC [22, 49, 50]. In our meta-analysis, the proportion of patients with LNM at each station was comparable between PPG and CDG. However, in previous reports, some patients with negative pathological lymph nodes (pN0) died of recurrence. Lymph node micrometastasis, a new concept, is suggested to be a cause of recurrent gastric cancers [17, 51, 52]. This concept refers to tumor cell clusters with tiny size or rare cells, which are considered pN0. The possibility of micrometastatic tumor cells in the remaining lymph nodes of No. 5 and No. 6 in vivo cannot be neglected in patients in whom PPG is performed [17].

The PRM and DRM are also important pathological indicators for PPG because the volume of the remnant stomach influences gastric function after surgery [43]. The present meta-analysis revealed that PPG had advantages in preserving more remnant stomachs than CDG. The distance from the lesion to the pylorus should be fully considered before surgery, as a short antral cuff length may lead to postoperative gastric stasis [18]. It is commonly believed that EGC located more than 4 cm away from the pyloric ring is an indispensable indication for PPG [9]. It is not surprising that the PPG group had a shorter distal margin than the CDG group because of pyloric ring preservation. Interestingly, our results revealed that surgeons also tend to retain more of the proximal stomach when performing PPG. Based on this, some studies attempted to widen the application of PPG for EGC, even involving “upper-third portion.” If a margin free of tumor can be achieved, PPG can be a substitute for total gastrectomy or subtotal gastrectomy with more desirable functional outcomes and lower postoperative morbidity [43].

CDG with LN dissection for gastric cancer generally provides a sufficiently satisfactory prognosis for EGC [53, 54]. In the KLASS-01 trial, a total of 1416 patients with EGC were randomly included. The 5-year OS rates were 94.2% in laparoscopic DG (*n* = 705) and 93.3% in open DG (*n* = 711) [53]. In comparison, there are few authoritative reports on the oncological outcomes of PPG. According to a Japanese multicenter propensity score-matched cohort analysis, the 5-year OS was 98.4% for the PPG group and 96.6% for the CDG group [39]. Zhu et al. reported that the 3-year RFS of the PPG group was similar to that of the CDG group (97.8% vs. 94.4%) [43]. The pooled results of the present meta-analysis suggested that PPG may have similar oncological outcomes compared with CDG. However, follow-up in the included studies was generally less than 5 years. Remnant gastric cancer, commonly diagnosed 10 to 30 years after initial surgery, could be an important oncological risk [55]. The regurgitation of bile or pancreatic juice has been thought to initiate carcinogenesis through mucosal damage in the remnant stomach [56]. The preservation of the pylorus ring during PPG would assuage gastritis by reducing the reflux of the duodenal contents. In this respect, the risk of remnant gastric cancer after PPG is supposed theoretically to be reduced.

There were certain limitations in our analysis. First, only two of the included studies were RCTs. This certainly attenuated the evidence level. Our results are inevitably impacted by the short follow-up duration and the limited numbers of patients in the included studies. The analysis of OS and RFS contained only three publications; the results of this study did not provide solid evidence of oncological safety. Second, PPG might be applicable only in countries with a high incidence of EGC such as East Asian countries. The results need further confirmation in other countries. Third, the uniform PPG procedure has not been completely established. The LN dissection nerve preservation and reconstructions may depend on institutional policies or the abilities of surgeons. Fourth, there were also inconsistencies among studies with respect to the standards used for patient inclusion.

## Conclusion

In conclusion, this meta-analysis revealed that fewer total lymph nodes were harvested in PPG than CDG. There were significant differences in the number of lymph nodes harvested at stations No. 5, No. 6, No. 9, and No. 11p. However, oncological outcomes, including OS and RFS, were comparable between the two procedures. In addition, our meta-analysis also found that PPG has shorter PRM and DRM, meaning that more remnant stomachs would be preserved in PPG. This may also be one of the reasons why PPG can improve postoperative function. Current findings are based mainly on observational studies, and adequately powered RCTs are required in the future.

## Supplementary Information

**Additional file 1.** Registration of the protocol in PROSPERO.

**Additional file 2.** The search strategy.

**Additional file 3.** The risk of bias among included studies. a. risk of bias of RCSs; b. risk of bias of RCTs.

**Additional file 4.** The pooled results of tumor size and histology. a. tumor size; b. differentiated; c. signet ring cell carcinoma.

**Additional file 5.** Funnel plots of publication bias. a. lymph node harvest; b. pathological T1a; c. pathological T1b; d. pathological N0; e. PRM.

## Data Availability

The datasets used and analyzed during the current study are available from the corresponding author upon reasonable request.

## Abbreviations

PPG: Pylorus-preserving gastrectomy
EGC: Early gastric cancer
CDG: Conventional distal gastrectomy
OR: Odds ratio
WMD: Weighted mean difference
CI: Confidence interval
SD: Standard deviation
LN: Lymph node
LNM: Lymph node metastasis
DRM: Distal resection margin
PRM: Proximal resection margin
OS: Overall survival
RFS: Recurrence-free survival
RCS: Retrospective cohort study
RCT: Randomized controlled trial
NOS: Newcastle–Ottawa Quality Assessment Scale
IPA: Infra-pyloric artery
