# Supplementary material for: Pathological and oncological outcomes of pylorus-preserving versus conventional distal gastrectomy in early gastric cancer: a systematic review and meta-analysis
Source: World J Surg Oncol. 2022 Sep 24;20:308. doi: 10.1186/s12957-022-02766-0 (PMC9508780; doi:10.1186/s12957-022-02766-0)
Supplement: Supplementary file 1 — Additional file 1. Registration of the protocol in PROSPERO. [file 12957_2022_2766_MOESM1_ESM.pdf]

To enable PROSPERO to focus on COVID-19 submissions, this registration record has undergone basic automated checks for eligibility and is published exactly as submitted. PROSPERO has never provided peer review, and usual checking by the PROSPERO team does not endorse content. Therefore, automatically published records should be treated as any other PROSPERO registration. Further detail is provided here.

Citation

Sen Hou, Zhi dong Gao, Yingjiang Ye. Pathological and oncological outcomes of pylorus preserving gastrectomy for early gastric cancer in the middle third : a systematic review with meta analysis. PROSPERO 2022 CRD42022304677 Available from:
https://www.crd.york.ac.uk/prospero/display_record.php?ID=CRD42022304677

Review question

Pylorus preserving gastrectomy (PPG)is a type of

function-preserving gastrectomy. Generally, patients are eligible for PPG when the gastric cancer is preoperatively determined to be a cT1cN0 tumor, when it involves a tumor located in the middle portion of the stomach. Comparative studies on PPG and distal gastrectomy (DG) have been
published and its advantages of reducing postgastrectomy syndromes have been proved. However, dissection of the lymph nodes of PPG is frequently incomplete, the distal resected margin was shorten theoretically. These may be worrisome in terms of oncologic safety. As the evidence of the evaluation of pathological and oncological outcomes of PPG were limited, we aimed to carry out this meta-analysis.

Searches

PubMed, Cochrane, Web of Science, and Embase will be searched. There were no language restrictions in our search.

Types of study to be included

Randomized controlled trials (RCTs) and retrospective cohort studies (RCSs) were included.Inclusion criteria: (1) studies included PPG group and DG group; (2) results including at least one outcome of interest.

Exclusion criteria: (1) less than 10 patients; (2) studies from the same institution or with overlapping patients;(3) animal experiments.

Condition or domain being studied

Pylorus preserving gastrectomy for early gastric cancer

Participants/population

Patients with a pathological diagnosis of primary EGC who were treated by radical gastrectomy and lymph node dissection.

Intervention(s), exposure(s)

PPG was performed with D1, D1+, or D2 lymph node dissection.

Comparator(s)/control

DG was performed with D1, D1+, or D2 lymph node dissection.

Main outcome(s)

Main outcomes: (1)pathological outcomes including the lymph nodes harvested, proximal resection margin, distal resection margin, tumor size, positive lymph node, lymphatic invasion, vascular invasion, histology, TNM stage, pyloric cuff;(2) oncological outcomes including death rate, overall survival, recurrence rate, recurrence free survival, etal.

Additional outcome(s)

None

Data extraction (selection and coding)

Two investigators independently will review the titles and abstracts of all citations identified by the literature

search. Eligible articles are reviewed for a duplicate in an independent manner by the two investigators.

Disagreement in data extraction is resolved by consensus.

Two authors independently extracted the following data: study demographics and characteristics, including: (1) first author, (2) publication year, (3) study design, (4) demographic of patients, (5) country, (6) multicenter or not, (7) date of inclusion, (8) tumor location,(9) pathological outcomes including the lymph nodes harvested, proximal resection margin, distal resection margin, tumor size, positive lymph node, lymphatic invasion, vascular invasion, histology, TNM stage, pyloric cuff;(2) oncological outcomes including death rate, overall survival, recurrence rate, recurrence free survival, etal.

Risk of bias (quality) assessment

Sensitivity analyses, cumulative analyses and subgroup analyses were conducted to investigate the influence on the overall results and discover the source of heterogeneity. Moreover, funnel plots, Harbord's test and Egger's test were performed to assess the publication bias of the included studies

Strategy for data synthesis

All statistical analyses will be conducted using the statistical software Stata (version 16)/Review Manager 5.4 . The mean

difference, standard deviation, and standard error are used for the meta-analysis.The OR was calculated along with its 95% CI for dichotomous outcomes and mean difference (MD) was calculated for continuous outcomes. Statistical heterogeneity among studies was assessed by means of ?2 and the extent of inconsistency was assessed by the I^2 statistic. The random-effects model and the fixed-effect model were used. If I^2 was less than 50% (cut-off point), we used the fixed-effect model, while if I^2 was more than 50%, we chose the random effects model. A rough guide to the interpretations of I^2 from the Cochrane Collaboration Handbook regards 0–40% as heterogeneity might not be important, 30–60% as moderate heterogeneity, and 75–100% as considerable heterogeneity. Sensitivity analyses, cumulative analyses and subgroup analyses were conducted to investigate the influence on the overall results and discover the source of heterogeneity. Moreover, funnel plots, Harbord's test and Egger's test were performed to assess the publication bias of the included studies

Analysis of subgroups or subsets

If heterogeneity exists?subgroup analysis will be performed according to the years of publication, country and districts, etc.

Contact details for further information

Sen Hou
housen95@163.com

Organisational affiliation of the review

Peking university people's hospital

Review team members and their organisational affiliations

Dr Sen Hou. Peking university people's hospital
Professor Zhi dong Gao. Peking university people's hospital
Professor Yingjiang Ye. Peking university people's hospital

Type and method of review

Intervention, Meta-analysis, Systematic review

Anticipated or actual start date

16 December 2021

Anticipated completion date
28 February 2022

Funding sources/sponsors
National Natural and Science Foundation of China (NNSFC)

Grant number(s)
State the funder, grant or award number and the date of award

81871962

Conflicts of interest

Language
English

Country
China

Stage of review
Review Ongoing

Subject index terms status
Subject indexing assigned by CRD

Subject index terms
MeSH headings have not been applied to this record

Date of registration in PROSPERO
20 February 2022

Date of first submission
20 January 2022

Stage of review at time of this submission
The review has not started

Stage	Started	Completed
Preliminary searches	No	No
Piloting of the study selection process	No	No
Formal screening of search results against eligibility criteria	No	No
Data extraction	No	No
Risk of bias (quality) assessment	No	No
Data analysis	No	No

The record owner confirms that the information they have supplied for this submission is accurate and complete and they understand that deliberate provision of inaccurate information or omission of data may be construed as scientific misconduct.
The record owner confirms that they will update the status of the review when it is completed and will add publication details in due course.

Versions

20 February 2022
20 February 2022
