# Supplementary material for: Pathological and oncological outcomes of pylorus-preserving versus conventional distal gastrectomy in early gastric cancer: a systematic review and meta-analysis
Source: World J Surg Oncol. 2022 Sep 24;20:308. doi: 10.1186/s12957-022-02766-0 (PMC9508780; doi:10.1186/s12957-022-02766-0)
Supplement: Supplementary file 2 — Additional file 2. The search strategy. [file 12957_2022_2766_MOESM2_ESM.pdf]

Search strategy for PubMed.

#1. “stomach neoplasms” [Mesh]

#2. “Neoplasm, Stomach” [Title/Abstract])

#3. “Stomach Neoplasm” [Title/Abstract])

#4. “Neoplasms, Stomach” [Title/Abstract])

#5. “Gastric Neoplasms” [Title/Abstract]

#6. “Neoplasm, Gastric” [Title/Abstract])

#7. “Neoplasms, Gastric” [Title/Abstract])

#8. “Cancer of Stomach” [Title/Abstract])

#9. “Stomach Cancers [Title/Abstract]

#10. “Gastric Cancer” [Title/Abstract]

#11. “Stomach Cancer” [Title/Abstract]

#12 “Cancer, Stomach” [Title/Abstract]

#13 “Cancer of the Stomach” [Title/Abstract]

#14 “Gastric Cancer, Familial Diffuse” [Title/Abstract]

#15. #1 OR #2 OR #3 OR #4 OR #5 OR #6 OR #7 OR #8 OR #9 OR #10 OR #11 OR #12 OR #13 OR #14

#16 “pylorus preserving gastrectomy” [MeSH])

#17 “PPG” [Title/Abstract]

#18. #16 OR #17

#19. #15 AND #18

Search strategy for EMBASE

#1. ('gastric cancer':ab,ti OR 'stomach neoplasms':ab,ti OR 'neoplasm, stomach':ab,ti OR 'gastric neoplasms':ab,ti OR 'gastric neoplasm':ab,ti OR 'neoplasm, gastric':ab,ti OR 'stomach neoplasm':ab,ti OR 'neoplasms, gastric':ab,ti OR 'cancer of stomach':ab,ti OR 'stomach cancers':ab,ti OR 'cancer, gastric':ab,ti OR 'cancers, gastric':ab,ti OR 'gastric cancers':ab,ti OR 'stomach cancer':ab,ti OR 'cancer, stomach':ab,ti OR 'cancers, stomach':ab,ti OR 'cancer of the stomach':ab,ti OR 'gastric cancer, familial diffuse':ab,ti) AND [1966-2022]/py

#2. (' pylorus preserving gastrectomy ':ab,ti OR 'PPG':ab,ti)AND [1966-2022]/py

#3. #1 AND #2

Search strategy for Cochrane and Web of science

The strategies were similar to that of PubMed.
