# Supplementary material for: Pathological and oncological outcomes of pylorus-preserving versus conventional distal gastrectomy in early gastric cancer: a systematic review and meta-analysis
Source: World J Surg Oncol. 2022 Sep 24;20:308. doi: 10.1186/s12957-022-02766-0 (PMC9508780; doi:10.1186/s12957-022-02766-0)

Additional file 3: Risk of bias of included studies

a. Risk of bias of RCSs

Study year	Study design	Selection	Comparability	Outcomes	NOS
Zhang 1998 [33]	RCS	☆ ☆ ☆	☆ ☆	☆ ☆	7
Kong 2009 [19]	RCS	☆ ☆ ☆	☆ ☆	☆ ☆	7
Ikeguchi 2010 [35]	RCS	☆ ☆ ☆	☆ ☆	☆ ☆ ☆	8
Kim 2014 [17]	RCS	☆ ☆ ☆	☆ ☆	☆ ☆	7
Suh 2014 [36]	RCS	☆ ☆ ☆	☆ ☆	☆ ☆ ☆	8
Hu 2015 [37]	RCS	☆ ☆ ☆	☆	☆ ☆	6
Hu 2016 [38]	RCS	☆ ☆ ☆	☆	☆ ☆ ☆	7
Aizawa 2017 [39]	RCS	☆ ☆ ☆	☆ ☆	☆ ☆ ☆	8
Hosoda 2017 [14]	RCS	☆ ☆ ☆	☆ ☆	☆ ☆ ☆	8
Xia 2018 [40]	RCS	☆ ☆ ☆	☆ ☆	☆ ☆	7
Eom 2019 [41]	RCS	☆ ☆ ☆	☆	☆ ☆ ☆	7
Xia 2019 [42]	RCS	☆ ☆ ☆	☆ ☆	☆ ☆	7
Zhu 2019 [43]	RCS	☆ ☆ ☆	☆ ☆	☆ ☆ ☆	8
Huang 2020 [44]	RCS	☆ ☆ ☆	☆ ☆	☆ ☆	7

b. Risk of bias of RCTs

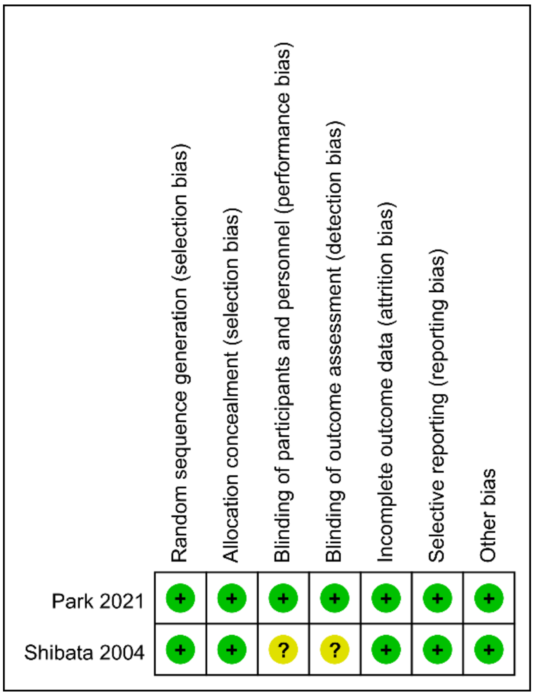

Supplement: Supplementary file 3 — Additional file 3. The risk of bias among included studies. a. risk of bias of RCSs; b. risk of bias of RCTs. [file 12957_2022_2766_MOESM3_ESM.pdf]
