# Supplementary material for: Pathological and oncological outcomes of pylorus-preserving versus conventional distal gastrectomy in early gastric cancer: a systematic review and meta-analysis
Source: World J Surg Oncol. 2022 Sep 24;20:308. doi: 10.1186/s12957-022-02766-0 (PMC9508780; doi:10.1186/s12957-022-02766-0)
Supplement: Supplementary file 4 — Additional file 4. The pooled results of tumor size and histology. a. tumor size; b. differentiated; c. signet ring cell carcinoma. [file 12957_2022_2766_MOESM4_ESM.pdf]

Additional file 4: Meta-analysis of tumor size and histology

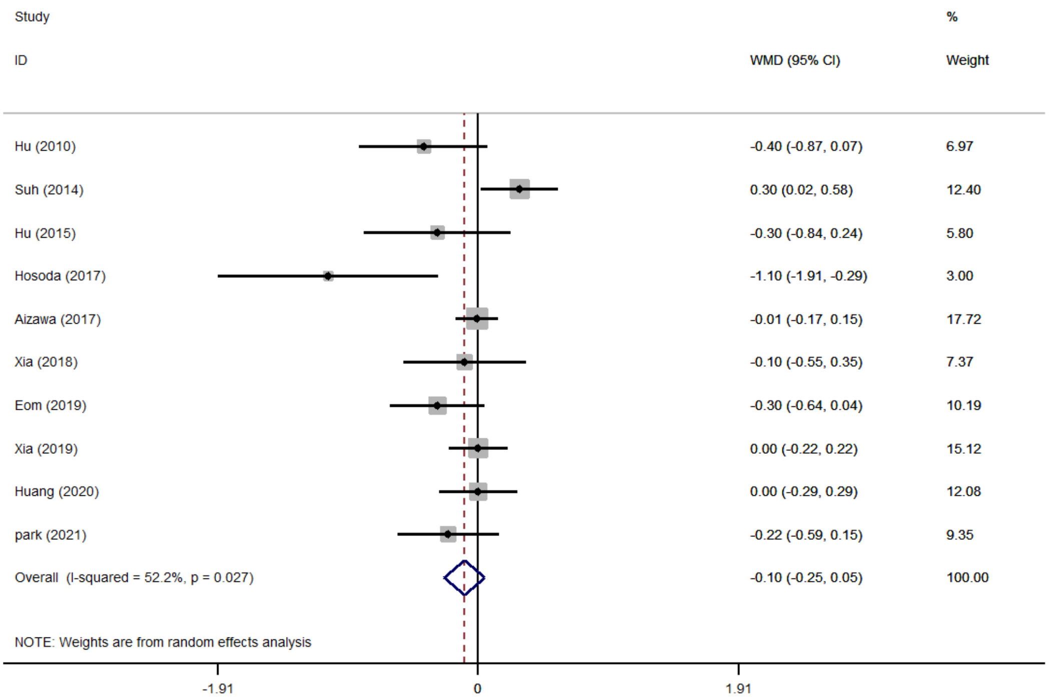

Additional file 4.1 Forest plots for the meta-analysis of tumor size.

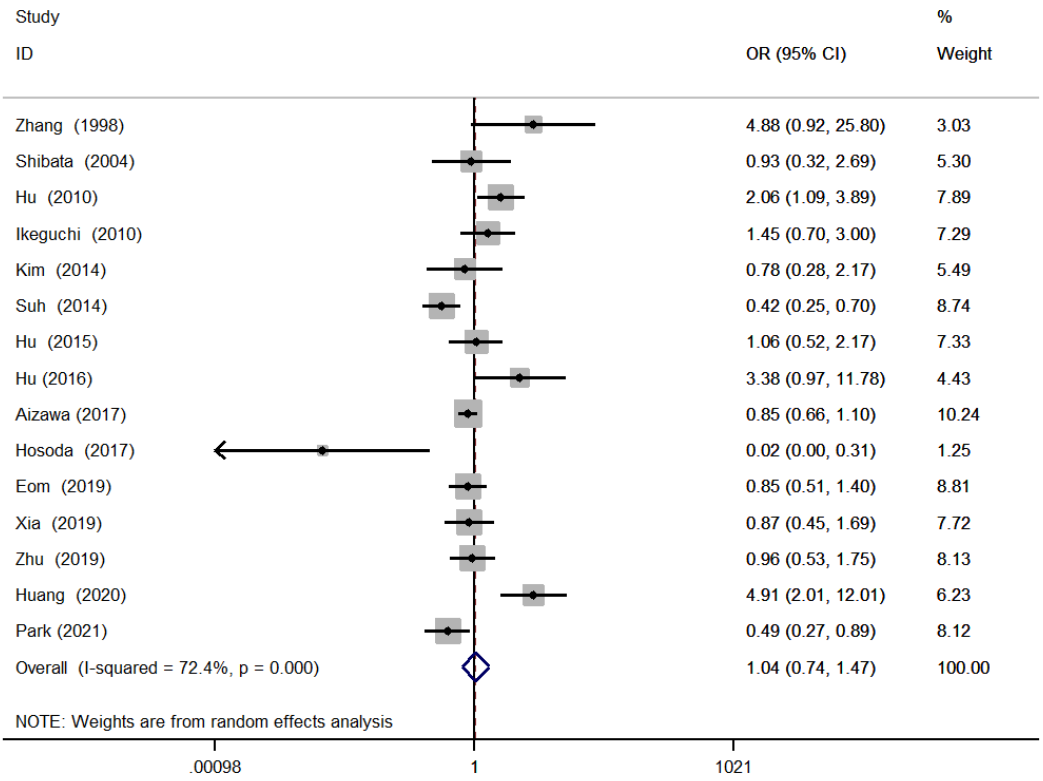

Additional file 4.2 Forest plots for the meta-analysis of differentiated carcinoma.

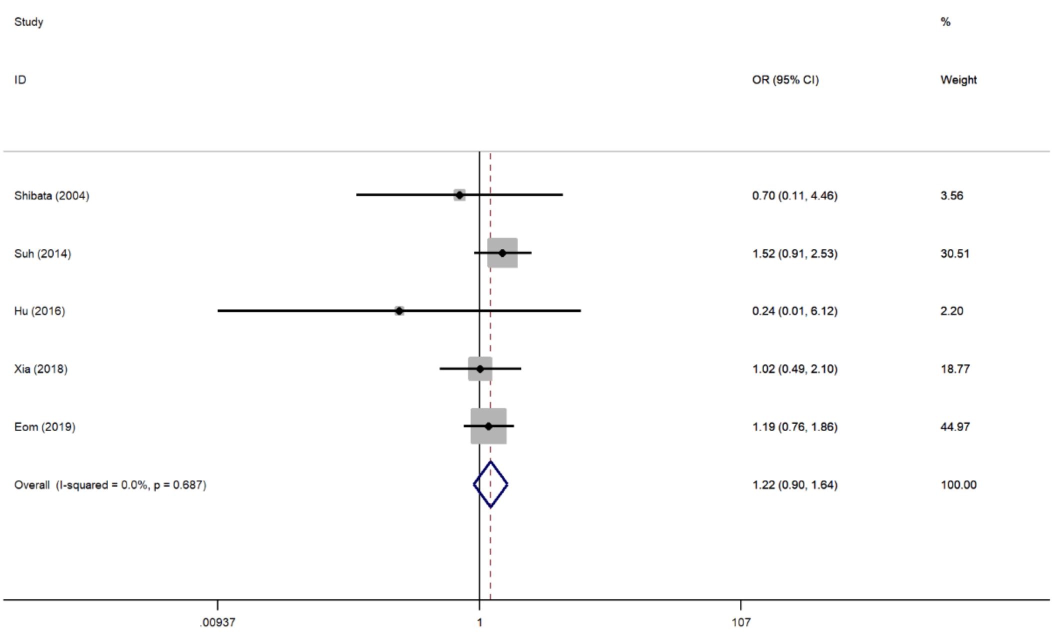

Additional file 4.3 Forest plots for the meta-analysis of signet ring cell carcinoma.
