# Supplementary material for: Pathological and oncological outcomes of pylorus-preserving versus conventional distal gastrectomy in early gastric cancer: a systematic review and meta-analysis
Source: World J Surg Oncol. 2022 Sep 24;20:308. doi: 10.1186/s12957-022-02766-0 (PMC9508780; doi:10.1186/s12957-022-02766-0)
Supplement: Supplementary file 5 — Additional file 5. Funnel plots of publication bias. a. lymph node harvest; b. pathological T1a; c. pathological T1b; d. pathological N0; e. PRM. [file 12957_2022_2766_MOESM5_ESM.pdf]

Additional file 4: Funnel plots of publication bias

a. Lymph node harvest

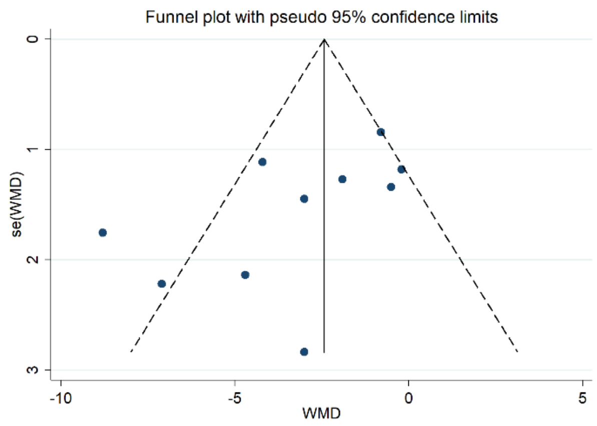

b. Pathological T1a

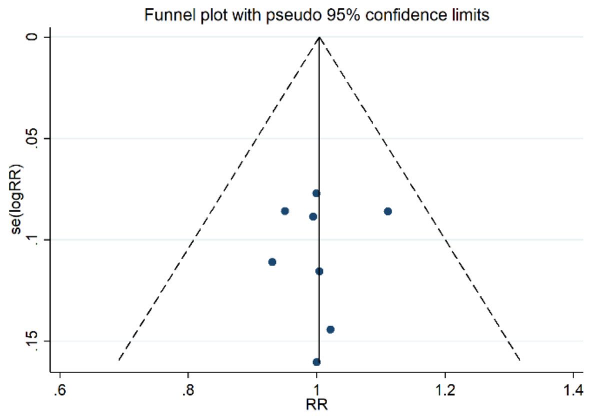

c. Pathological T1b

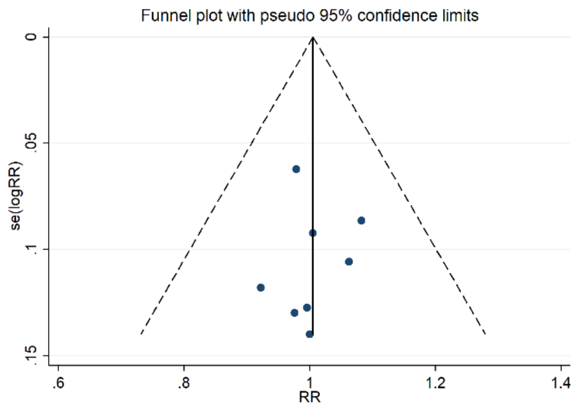

d. Pathological N0

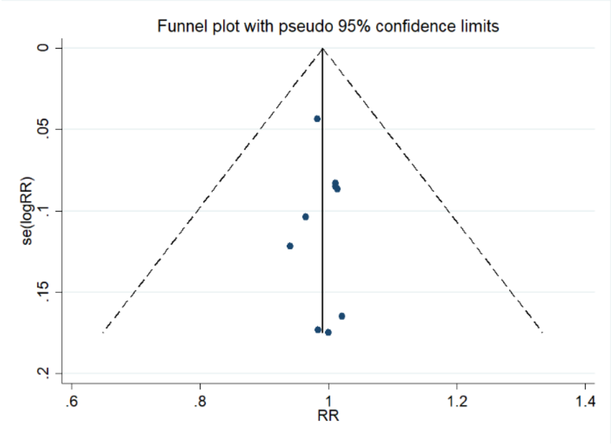
Funnel plot with pseudo 95% confidence limits
se(logRR)
0
.05
.1
.15
.2
.6
.8
1
1.2
1.4
RR

e. PRM

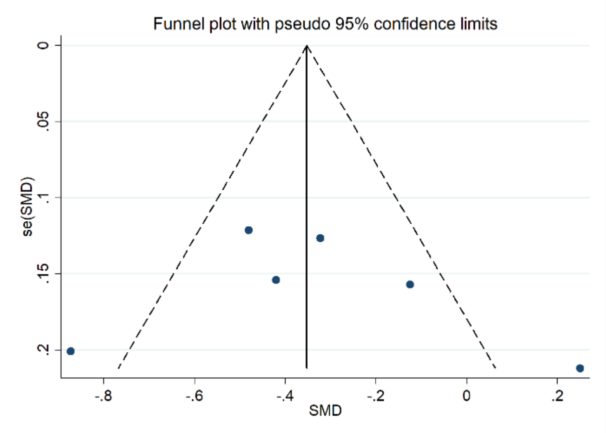
Funnel plot with pseudo 95% confidence limits
se(SMD)
0
.05
.1
.15
.2
-.8
-.6
-.4
-.2
0
.2
SMD
